# Embryo Cryopreservation: What do couples think about it?

**DOI:** 10.5935/1518-0557.20240093

**Published:** 2025

**Authors:** Julia Picinato Medeiros de Araujo Rocha, Carolina Gennari Verruma, Ana Luiza Camargos Morato, Maria Cristina Picinato, Rui Alberto Ferriani, Rosana Maria dos Reis

**Affiliations:** 1 Department of Gynecology and Obstetrics, Ribeirão Preto Medical School of University of Sao Paulo, Ribeirão Preto, Brazil

**Keywords:** surplus embryos, embryo donation, bio-rights, assisted reproductive technologies

## Abstract

**Objective:**

To investigate the perspectives of infertile couples regarding embryo cryopreservation throughout assisted reproduction treatment.

**Methods:**

The convenience sample included infertile couples undergoing assisted reproduction treatment. They responded to a questionnaire specifically designed to gauge views and opinions on cryopreservation of surplus embryos. Statistical analysis was conducted using SPSS, employing the Mann-Whitney U and Fisher’s exact tests (p<0.05).

**Results:**

The study included 187 couples, with average ages of 35 years for women and 38 years for men. A total of 182 couples (97.3%) agreed with the practice of freezing surplus embryos and, the desire to increase the odds of pregnancy within the same ovarian stimulation cycle was the main motivation (89%). Almost 40% of participants said they might consider embryo donation to other couples for assisted reproductive treatment. However, less than 20% of volunteers (15.38% of women and 12.08% of men) expressed the desire to donate for research purposes. Women with higher levels of education were significantly (*p*<0.05) more likely to support the donation of surplus embryos for research. Gender, religion, and education did not influence (*p*>0.05) the respondents’ perspectives regarding the beginning of life and posthumous conception.

**Conclusions:**

Embryo cryopreservation provides infertile couples with hope however, the fate of surplus embryos remains controversial. A careful approach with appropriate regulation is necessary to ensure safe and ethical practices.

## INTRODUCTION

Assisted reproductive technologies (ARTs) primarily aim to assist infertile couples in achieving the goal of having a healthy baby (Salcuni *et al.*, 2021). However, within this context, challenging scenarios continuously arise due to societal pressures and evolving needs. These include but are not limited to, the desire for conceiving children in diverse family structures, the need for fertility preservation in cases of cancer or serious illness, and the aspiration for the births of healthy baby in couples with genetic conditions (Dolmans & Manavella, 2019; Fauser *et al.*, 2019; Bayefsky *et al.*, 2024; Van Steijvoort *et al.*, 2024). In a broader sense, technological advancements in ARTs may give rise to ethical, legal, and social implications, as established standards often lag behind increasing demands (Araujo & Araujo, 2018).

As part of ART procedures, it is common for couples the use of gamete and embryo cryopreservation to increase the odds of pregnancy (Araujo & Araujo, 2018). The technique of cryopreservation involves the preservation of cells and/or tissues at low temperatures. It serves as a valuable preservation method when surplus embryos are available or when fresh embryo transfer is not feasible (Jang *et al.*, 2017; Rienzi *et al.*, 2018). Similarly to gametes, embryos can remain cryopreserved for prolonged periods and be thawed for further attempts. According to the Brazilian Health Regulatory Agency (*Agência Nacional de Vigilância Sanitária* - ANVISA), around 200,000 embryos were frozen in both public and private clinics between 2020 and 2021 in Brazil (Anvisa, 2022).

When couples succeed in starting a family or have given up trying to have a baby, the good-quality embryos have three main options: donation to other couples, research, and discard (Simopoulou et al., 2019). This decision raises questions concerning each couple’s rights and duties, from the moment they approve cryopreservation by signing informed consent (*Termo de Consentimento Livre e Esclarecido* - TCLE), designed in agreement with Biolaw principles, which usually occurs before treatment commences (Silva *et al*., 2017).

Couple’s desire must be expressly stated in the TCLE, particularly in cases of divorce or the death of one of the spouses (Estanislau Gasda, 2015) and, current evidence suggests that major technological advances have ultimately created many surplus embryos. This could become a challenge for fertility clinics around the world as the years go by. In addition, the laws on assisted reproductive treatments are lagging behind, and one of the major ethical issues is the moral status of the human embryo (Bartolacci *et al.*, 2024).

Considering the increasing legal demands, tremendous improvement is needed. In particular, the current laws need to be amended appropriately and in a timely manner. Therefore, the aim of this study was to evaluate the perspectives of infertile couples regarding cryopreservation and possible donation of surplus embryos throughout the course of assisted reproduction treatment.

## MATERIALS AND METHODS

This convenience sampling protocol was approved by the Research Ethics Committee (CAAE: 68634917.2.0000.5440) of the Ribeirão Preto Medical School, University of São Paulo (*Hospital das Clínicas da Faculdade de Medicina de Ribeirão Preto da Universidade de São Paulo* - HCFMRP-USP). The study included couples who had been diagnosed with infertility and undergoing assisted reproduction treatment at the Human Reproduction Center of the HCFMRP-USP, during the period from January 2019 to March 2020. Participating couples were selected inclusively, without regard to ethnicity, education, or socioeconomic status. Data collected were analyzed anonymously.

**Supplementary figure 1:**
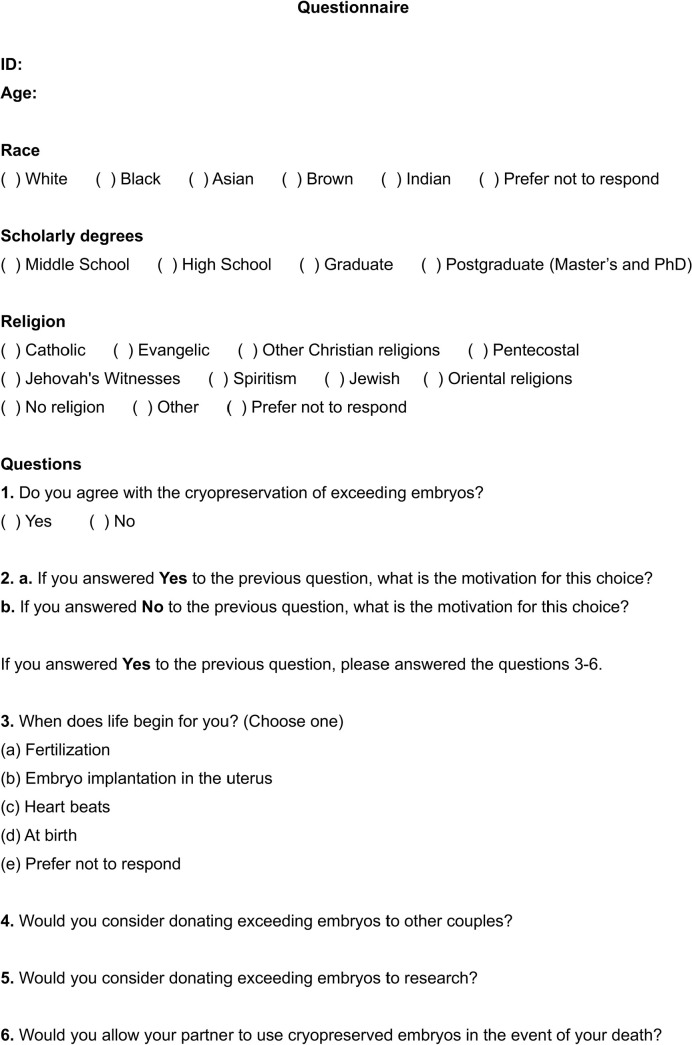
Questionnaire applied to volunteers.

After obtaining informed consent (signed TCLE), the participating couples completed a questionnaire. This questionnaire comprised five multiple-choice questions and four open-ended questions pertaining to cryopreservation and the handling of surplus embryos (Supplementary Figure 1). Additionally, the questionnaire included questions regarding their views about rights and duties and how they address these matters. There was no request for justification in the open-ended questions.

### Statistical Analysis

Continuous variables were compared using the Mann-Whitney U test, while categorical variables were analyzed using Fisher’s exact test. All statistical analyses were conducted using SPSS^®^ software, with a significance level of 0.05.

## RESULTS

**Figure 1:**
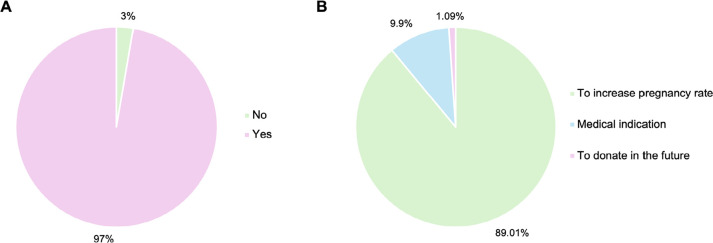
Volunteers’ views on cryopreservation of surplus embryos. (A) Couples who agreed (pink) or disagreed (green) to cryopreservation of surplus embryos. (B) The reasons why they agreed to cryopreserve surplus embryos.

The study included 187 couples (187 women and 187 men) who were undergoing assisted reproduction treatment. The characteristics of the volunteers are described in Table 1. Among the 187 couples interviewed, an overwhelming majority of 182 (97.3%) agreed to cryopreserve their surplus embryos (Figure 1A). When asked about their motivation for embryo cryopreserving, 162 couples (89.01%) cited their desire to enhance the likelihood of achieving pregnancy within a single ovarian stimulation cycle (Figure 1B).

**Table 1:** Clinical characteristics, scholarly degree, and religion of the 187 volunteers.

Characteristics	Women	Men
**Age - Mean [Mean (±SD)]**	35.1 (± 4.05)	38.9 (± 5.70)
**Race [n (%)]** White Asian Brown Black Indian Did not declare	132 (70.6%) 46 (24.6%) 1 (0.50%) 7 (3.70%) 1 (0.50%) 0 (0%)	121 (64.7%) 9 (4.84%) 29 (15.50%) 2 (1.06%) 0 (0%) 26 (13.90%)
**Scholarly degree [n (%)]** Postgraduate Graduate High School Middle School Did not declare	59 (31.60%) 60 (32%) 63 (33.70%) 5 (2.70%) 0 (0%)	31 (16.58%) 81 (43.32%) 44 (23.53%) 6 (3.20%) 25 (13.36%)
**Religion [n (%)]** Catholic Evangelic Spiritism Other	146 (78.1%) 27 (14.43%) 10 (5.34%) 4 (2.13%)	7 120 (64.17%) 27 (14.43%) (3.74%) (4.28%)

SD, standard deviation; n, number of volunteers.

**Figure 2:**
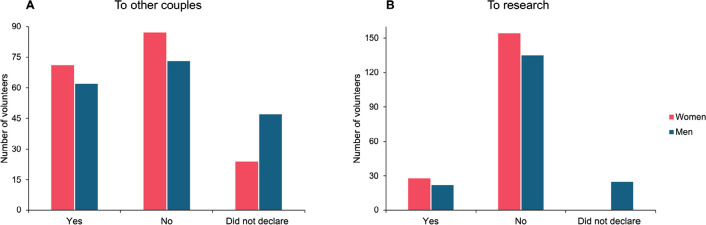
Male *vs*. female perspectives on donating surplus embryos (A) to other couples and (B) to research.

When asked about their willingness to donate embryos to other couples, less than 40% of respondents [71 women (39.01%) and 62 men (34.07%)] indicated that they would consider such a donation. Conversely, the majority either expressed reluctance to donate embryos or opted not to respond (Figure 2A). When stratified by scholarity and religion, no significant differences (*p*>0.05) were observed (Table 2).

**Table 2:** Volunteer views regarding embryo donation to other couples, research and use of embryo in case of death of one partner. The volunteers were classified according to scholarly degree and religion.

Question		Women^*^ [n (%)]		p value	Men^*^ [n (%)]		p value
			
		**Yes**	**No**		**Yes**	**No**	
**Donation to other couples**	**Scholarly degree** Graduate High School Middle School	47 (47.0%) 22 (40.0%) 2 (50.0%)	53 (53.0%) 33 (60.0%) 2 (50.0%)	0.661	43 (47.8%) 17 (41.5%) 2 (50.0%)	47 (52.2%) 24 (58.5%) 2 (50.0%)	0.839
	**Religion** Catholic Evangelic Spiritism Other	54 (42.8%) 13 (56.5%) 3 (42.9%) 1 (50.0%)	72 (57.2%) 10 (43.5%) 4 (57.1%) 1 (50.0%)	0.687	43 (42.1%) 14 (60.9%) 1 (25.0%) 3 (50.0%)	59 (57.9%) 9 (39.1%) 3 (75.0%) 3 (50.0%)	0.332
**Donation to research**	**Scholarly degree** Graduate High School Middle School	25 (21.1%) 3 (5.3%) 0 (0.0%)	93 (78.9%) 56 (94.6%) 5 (100.0%)	**0.010**	15 (13.7%) 6 (18.2%) 1 (20.0%)	94 (86.3%) 27 (81.8%) 4 (80.0%)	0.741
	**Religion** Catholic Evangelic Spiritism Other	24 (16.6%) 3 (12.0%) 1 (11.1%) 0 (0.0%)	120 (83.4%) 22 (88.0%) 8 (88.9%) 4 (100.0%)	0.975	17 (14.5%) 3 (11.5%) 1 (16.7%) 1 (12.5%)	100 (85.5%) 23 (88.5%) 5 (83.3%) 7 (87.5%)	0.976
**Use of embryo postmortem**	**Scholarly degree** Graduate High School Middle School	91 (81.2%) 44 (77.2%) 3 (60.0%)	21 (18.8%) 13 (22.8%) 2 (40.0%)	0.344	87 (82.8%) 34 (87.2%) 3 (60.0%)	18 (17.2%) 5 (12.8%) 2 (40.0%)	0.241
	**Religion** Catholic Evangelic Spiritism Other	105 (77.2%) 22 (88.0%) 8 (88.9%) 3 (75.0%)	31 (22.8%) 3 (12.0%) 1 (11.1%) 1 (25.0%)	0.562	90 (81.8%) 22 (84.6%) 4 (80.0%) 7 (87.5%)	20 (18.2%) 4 (14.5%) 1 (20.0%) 1 (12.5%)	0.964

* The analysis was performed only with subjects who answered yes or no. Those who did not answer were excluded. In bold, *p*<0.05; n, number of volunteers.

When asked about donating embryos for research, only 28 (15.38%) women and 22 (12.08%) men said they would consider donating their cryopreserved embryos for research. (Figure 2B). Most of women (n=25; 89.3%) who stated that they would donate their surplus embryos to research were college graduates (*p*=0.01), while the remaining (n=3; 10.7%) were high school graduates (Table 2). Among men who supported surplus embryo donation for research purposes, education did not impact their decision. Religion did not influence willingness to donate (or not to donate) surplus embryos for research in either women or men (Table 2).

**Figure 3:**
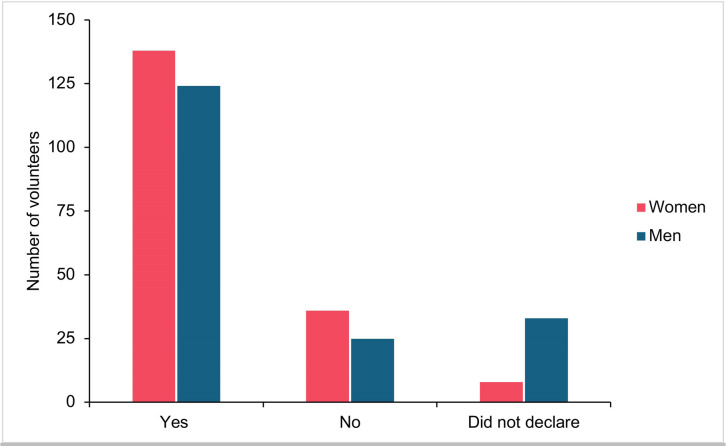
Male *vs*. female perspectives on posthumous assisted reproduction.

Concerning posthumous conception, most of the volunteers, more specifically 138 women (75.8%) and 124 men (68.1%), were in favor of this practice (Figure 3), irrespective of gender, education, or religion (Table 2).

**Figure 4:**
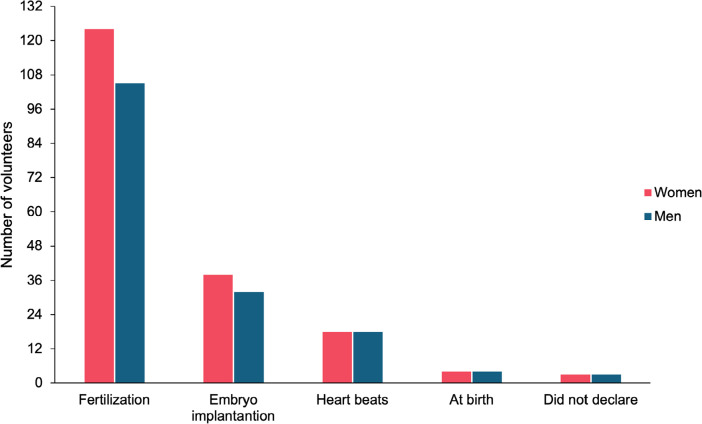
Volunteers’ perspectives on the beginning of life.

Furthermore, most respondents considered that life begins at fertilization of oocyte by sperm (124 women and 105 men), followed by embryo implantation in the uterine wall, heartbeat detection, and birth, regardless of gender, education, or religion (Figure 4).

## DISCUSSION

Cryopreservation of human embryos stands as a significant avenue for infertile couples seeking to achieve their aspirations of parenthood. In this study, 187 couples undergoing infertility treatment were questioned about their views on embryo cryopreservation, donation and postmortem use. The findings revealed a noteworthy trend: most couples were in favor of surplus embryo cryopreservation, regardless of education, religion, or socioeconomic status.

During assisted reproduction cycles, it is not uncommon for couples to have multiple embryos suitable for transfer. However, there are concerns about multiple pregnancy and its complications, such as gestational hypertension, pre-eclampsia, and preterm delivery (Henry *et al.*, 2013; Narang & Szymanski, 2020; Chen *et al.*, 2022). In that scenario, the embryo cryopreservation allows couples to transfer a safe number of embryos, usually one or two, to the uterus, while preserving the surplus embryo for indetermined period. These embryos can be used later, without a significant adverse impact on survival, implantation, and embryonic development (Loutradi *et al.*, 2008; Schiewe & Anderson, 2017; Nagy *et al.*, 2020). Cryopreservation has made it possible to establish oocyte and sperm banks, especially for those who wish to postpone motherhood or who are undergoing aggressive clinical treatment such as chemo and/or radiotherapy (Alikani, 2018; Bayefsky *et al.*, 2024; Ethics Committee of the American Society for Reproductive Medicine, 2024).

In our study, 97% of the couples supported cryopreservation of their surplus embryos at the end of the assisted reproduction treatment for various reasons. The majority believed that cryopreserving surplus embryos could increase the chances of pregnancy in the same ovarian stimulation cycle. In recent years, the number of embryos cryopreserved in public and private clinics had significantly increased in Brazil. This number increased 225% over 10 years, from 32,181 in 2012 to 114,372 in 2022 (Anvisa, 2022).

Although cryopreservation has made it possible to store embryos for future use, the technique has also raised concerns about the fate of surplus embryos. Cryopreserved embryos can be stored for decades for a fee, but they can also be donated or, in some cases, discarded. Christianson *et al*. (2020) reported that more than 1 million embryos are likely to be cryopreserved in the United States of America. In numerous cases, couples simply abandon their surplus embryos after successfully achieving their desired parenthood goal (Klock & Lindheim, 2022).

In our study, less than 40% of volunteers revealed that they would consider donating their surplus embryos to other couples. However, in practice, the numbers differ, and the number of cryopreserved embryos donated to other couples is very low. Recent studies conducted in the United States of America and United Kingdom reported that less than 2% of embryo transfers were from donated embryos (Lee *et al.*, 2023; Yue & MacKellar, 2024).

The number of embryos donated to research may be even lower. In our survey, fewer than 16% of women and 13% of men affirmed that they would consider donating embryos to research. Our results suggest that educational attainment influences women’s decisions, as the majority of those who expressed a willingness to consider embryo donation were university graduates. This low percentage is consistent with other studies. Klock and Lindheim (Klock & Lindheim, 2022) revealed that approximately 10% of couples who underwent assisted reproduction consider donating surplus embryos. In Brazil, the donation to research is even lower, only 63 embryos were donated to research between 2020 and 2023 (Anvisa, 2024). According to Scully *et al*. (2012), the donation of surplus embryos for research has more to do with the moral status of the embryo, which is not considered a living being by the donating couples, than with gratitude or indebtedness to ARTs.

In addition to the choices that couples face regarding surplus embryos once they no longer intend to use them, some choices must be made even before they start assisted reproduction treatment. These decisions include defining the legal status of embryos, determining their rights, and establishing the rights that should be credited by the legal system (Branco, 2009). Each country has its own laws regarding the ARTs, and Brazil is no exception (Klock & Lindheim, 2022). Several laws, such as 1184/2003, 11105/2005 and 2320/2022, help patients, professionals and fertility clinics regarding the manipulation, storage and use of gametes and embryos (Câmara dos Deputados, 2003; Congresso Nacional, 2005; CFM, 2022) The current legislation fails to address all potential issues and conflicts concerning cryopreserved embryos. For example, when both spouses die, or when one person wishes to use the cryopreserved embryos following divorce. Consequently, these gaps in the legal system must be bridged to provide both healthcare professionals and patients with greater clarity, safety, and effective decision-making guidance.

One factor that may influence the decision to donate surplus embryos is the belief of when life begins. The thought that it begins during conception could make the decision to donate an embryo for research more difficult (Paulson, 2022; Adamczyk *et al.*, 2024). When questioned about when life begins, most volunteers stated that life begins at fertilization, followed by moment of the embryo implantation in the uterus wall. In line with our results, a North American study that included different scholarly degree showed that most respondents also indicated that life started at conception (Jacobs, 2018).

Although the terms fertilization and conception are often used as synonymous, they do not mean the same thing. Fertilization is the fusion of the pronucleus of the sperm and the oocyte, while the term conception is much more complex and goes beyond fertilization, since the development of the embryo depends, among other things, on its successful implantation on the uterine wall (Rivera López, 2013; Zegers-Hochschild *et al.*, 2013). Feinberg *et al*. (2024) mentioned that the use of the term conception is more religious than scientific and, cryopreserved embryos cannot be treated as living beings. However, after a recent incident which resulted in the loss of cryopreserved embryos, the Alabama Supreme Court issued a ruling declaring that embryos created through *in vitro* fertilization should be considered children (Tanne, 2024).

This perspective on the commencement of life resonates with the stance adopted by the American Society for Reproductive Medicine (ASRM). According to the ASRM, the embryo merits a distinct status compared to other cellular entities, as it represents a potential life but should not be afforded the same level of protection as a living being (Ethics Committee of the American Society for Reproductive Medicine, 2009). The complexity of the topic is also observed in the laws that vary from country to country. For example, according to Article 2 of the Brazilian Civil Code, legal personality begins at live birth, but the law also safeguards the rights of the unborn child from the moment of conception (Brazil, 2002).

Religion emerges as another potentially factor that could be relevant when deciding to cryopreserve embryos. In our study, a majority of respondents stated that they were Roman Catholic, reflecting the world population since Catholicism is the predominant religion worldwide (Sallam & Sallam, 2016; Ipsos Global Advisor Lead, 2023). Some members of the Catholic Church oppose the use of ARTs, including cryopreservation (Schenker, 2005). In a study on patients’ preferences in completing assisted reproduction treatment, Alexander *et al*. (2020) showed that most women/couples who opted to donate their embryos, particularly for research purposes, were either unaffiliated with any religion or chose to abstain from answering questions pertaining to religious affiliation. Moreover, Kerridge *et al*. (2010) showed that embryo donation for research purposes could be a problematic issue for the Catholic and Buddhist communities.

In our investigation, couples’ views about embryo use when one of the partners dies were also discussed. Remarkably, irrespective of gender, religion, or education, most couples agreed to the use of cryopreserved embryos under such circumstances. In Brazil, Law 1.851/2022 was enacted, allowing the post-mortem use of cryopreserved embryos from couples who have undergone ART together. It also requires fertility clinics to ask couples about this alternative before starting treatment (Senado Federal, 2022). Cases such as these require a careful approach to ensure appropriate use in terms of safety and ethics.

Across different nations, attitudes toward postmortem gamete and embryo use vary. Although posthumous conception is banned in France, it is conditionally allowed in the United Kingdom and Brazil (Barać, 2023). Recognizing these international disparities, the ASRM advocates for fertility clinics to establish clear policies, especially regarding procedures that may be performed in different cases of posthumous conception scenarios (Ethics Committee of the American Society for Reproductive Medicine, 2018).

The timing of administering the questionnaire at the outset of assisted reproduction treatment presents a potential limitation to our study, as it precludes the assessment of how many couples ultimately opt to cryopreserved surplus embryos and their perspectives on the future of those embryos. Nevertheless, the study population was selected to include couples from diverse socioeconomic backgrounds, regardless of education and religion, so that the sample was representative of our population since assisted reproduction treatment is available in both public and private in our health service.

Human embryo cryopreservation is widely embraced by infertile couples, primarily to increase the odds of achieving pregnancy within a single cycle of assisted reproduction treatment. In our population, the fate of cryopreserved surplus embryos was not influenced by education, religion, or ethnicity. However, we did observe a positive correlation between women’s level of education and their decision to donate embryos for research. While embryo cryopreservation offers promising prospects for infertile couples, the fate of surplus embryos remains controversial.
